# On a limitation of Zeeman polarimetry and imperfect instrumentation in representing solar magnetic fields with weaker polarization signal

**DOI:** 10.1051/swsc/2021003

**Published:** 2021-02-12

**Authors:** A.A. Pevtsov, Y. Liu, I. Virtanen, L. Bertello, K. Mursula, K.D. Leka, A.L.H. Hughes

**Affiliations:** 1 National Solar Observatory, 3665 Discovery Drive, 3rd Floor, Boulder, CO 80303, USA; 2 Stanford University, Stanford, CA 94305-4085, USA; 3 ReSoLVE Centre of Excellence, Space Climate Research Unit, University of Oulu, POB 3000, 90014 Oulu, Finland; 4 NorthWest Research Associates, 3380 Mitchell Lane, Boulder, CO 80301, USA; 5 Institute for Space-Earth Environmental Research, Nagoya University, Furo-cho Chikusa-ku Nagoya, 464-8601 Aichi, Japan

**Keywords:** Sun: magnetic fields, techniques: polarimetric

## Abstract

Full disk vector magnetic fields are used widely for developing better understanding of large-scale structure, morphology, and patterns of the solar magnetic field. The data are also important for modeling various solar phenomena. However, observations of vector magnetic fields have one important limitation that may affect the determination of the true magnetic field orientation. This limitation stems from our ability to interpret the differing character of the Zeeman polarization signals which arise from the photospheric line-of-sight vs. the transverse components of the solar vector magnetic field, and is likely exacerbated by unresolved structure (non-unity fill fraction) as well as the disambiguation of the 180° degeneracy in the transverse-field azimuth. Here we provide a description of this phenomenon, and discuss issues, which require additional investigation.

## 1 Introduction

Observations of magnetic fields provide key information for developing our understanding of the Sun’s short-term (space weather) and long-term (space climate) activity and in predicting these effects on Earth. Synoptic full disk longitudinal magnetograms have existed since the late 1960s, and these data continue to serve as the primary input for space weather and space climate research and operational forecasts. By their nature, longitudinal magnetograms do not contain sufficient information to derive the true orientations of the magnetic-field vectors, and thus, require additional assumptions for physical interpretation. For example, “radial field” synoptic maps, which are widely used in space weather forecasting are created under the assumption that the true field is radial. Observations of vector Stokes polarimetery in principle have the information necessary to fully reconstruct photospheric vector-magnetic-field maps, and efforts are underway to employ such data in operational space weather forecasts.

The earliest observations of vector magnetic fields in solar active regions were conducted at the Crimean Astrophysical Observatory in the early 1960s (Stepanov & Severny, 1962; Severny, 1965). By the early 1980s, a number of vector magnetographs were developed around the world, with the most prolific instruments operating in Czechoslovakia, East Germany (Pflug & Grigoryev, 1986), Japan (NAOJ, Ichimoto et al., 1993), the Soviet Union (Crimean, Pulkovo and Sayan observatories), and the USA (NASA’s Marshall Space Flight Center/MSFC, Mees Solar Observatory of University of Hawaii, High Altitude Observatory/HAO) (for review, see individual articles in Hagyard, 1985; Cacciani et al., 1990). All of these instruments had a limited field of view, typically about the size of an average active region. Full disk vector magnetograms have been routinely observed since late 2003 by the Vector Stokes Magnetograph (VSM) on the Synoptic Optical Long-term Investigation of the Sun (SOLIS) platform (Keller et al., 2003). Beginning in 2010, full disk vector magnetograms are available from the Helioseismic and Magnetic Imager (HMI, Scherrer et al., 2012) on board the Solar Dynamics Observatory (SDO).

**Fig. 1.**
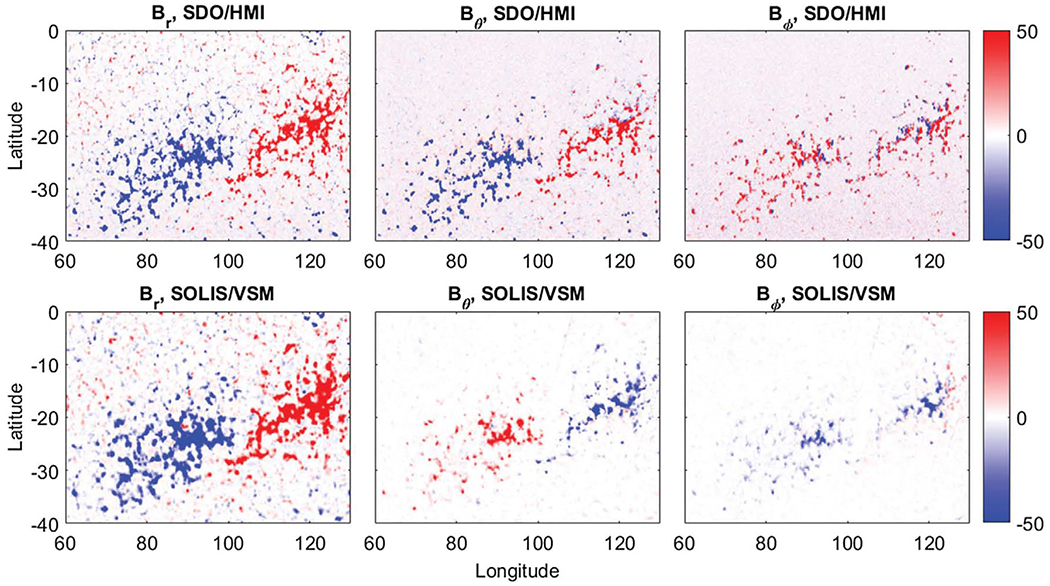
Example of disagreement in orientation of *B*_*θ*_ and *B*_*φ*_ Magnetic field vector components of an active region in the southern hemisphere, which passed the central meridian on 19 November 2015. Upper panels show radial (*B*_*r*_), meridional (*B_θ_*) and zonal (*B*_φ_) field from SDO/HMI and lower panels from SOLIS/VSM. Meridional (middle) and zonal (right) components depict opposite orientation. Red/blue represent positive/negative polarities scaled between ±50 G (see color bars on the right side of figure).

On 23–26 January 2017, a working meeting on the “Use of Vector Synoptic Maps for Modeling” was held in Oulu, Finland with two follow up meetings at the National Solar Observatory, Boulder, CO, USA (7–10 November 2017), and at the Max Planck Institute for Solar System Research, Göttingen, Germany (18–21 September, 2018). At the first meeting, a direct comparison of vector magnetic field observations from SDO/HMI and SOLIS/VSM revealed disagreements in the orientation of meridional (*B_θ_*, North–South) and/or zonal (*B_φ_*, East–West) components of the vector field in plage areas. One example of this disagreement is shown in Figure 1, which shows the three components of the magnetic field in VSM and HMI synoptic maps between heliographic latitudes −40° and 0° and Carrington longitudes 60° and 130°.

This decaying active region (plage) was at the central meridian on 19 November 2015. A synoptic map is used as an example because the averaging used in producing a synoptic map results in lower background noise, but very similar patterns are also seen in less-averaged full-disk data. Recent comparisons also indicate that the average large-scale zonal field *B_φ_* observed by SOLIS/VSM and derived from line-of-sight observations tends to disagree outside active regions (Virtanen et al., 2019). The investigation of this disagreement uncovered what we think may be an important limitation to Zeeman vector polarimetry. Here we present the results of our investigation into the discrepancies uncovered by that collaborative effort. In Section 2, we introduce observations from two vector magnetographs and compare the vector field derived from these instruments and their standard data reduction algorithms. Sections 3 and 4 introduce our explanation of the observed disagreement, and in Section 5 we discuss the results of our findings.

## 2 Vector magnetograms from different instruments and a comparative analysis

Here, we employ full disk vector magnetograms from two instruments: the Vector Stokes Magnetograph (VSM) on the Synoptic Optical Long-term Investigations of the Sun (SOLIS) platform (Keller et al., 2003; Balasubramaniam & Pevtsov, 2011), and the Helioseismic and Magnetic Imager (HMI, Scherrer et al., 2012; Hoeksema et al., 2014) on board the Solar Dynamics Observatory (SDO, Pesnell et al., 2012).

The HMI instrument is a filtergraph covering the full solar disk with 4096 × 4096 pixels. The spatial resolution is about 1″ with a 0.5″ pixel size. The width of the filter profiles is 7.6 pm. The spectral line used is Fe i 617.3 nm, which forms in the photosphere (Norton et al., 2006). The Stokes parameters (*I*, *Q*, *U*, *V*) are computed from those measurements (Couvidat et al., 2016), and are further inverted to retrieve the vector magnetic field using a Milne-Eddington (ME) based inversion algorithm, the Very Fast Inversion of the Stokes Vector (VFISV Borrero et al., 2011; Centeno et al., 2014). To suppress *p*-mode oscillations and to increase the signal-to-noise ratio, registered filtergrams are averaged over a certain time before computing the Stokes vector. Currently a weighted average is computed every 720 s using data obtained over 1350 seconds by default; other averaging windows are available.

Inversion of the vector field has an unavoidable 180° ambiguity in the azimuthal field direction. Assumptions about the field must be made to resolve the ambiguity. For all pixels in active regions, as well as for strong-field pixels (where the *S/N* > 3 in the transverse signal plus a 50 G buffer) in quiet Sun regions, the azimuth is determined using a minimum energy algorithm (Metcalf, 1994; Metcalf et al., 2006; Leka et al., 2009; Hoeksema et al., 2014). The minimum-energy-method computation is time consuming for pixels where the signal is dominated by noise, so for weaker polarization regions, the 180° ambiguity is solved using three quicker methods: a randomizing method (the option to add 180° is determined randomly), an acute-angle comparison to a potential field, and a method that provides the most radially-directed solution. More details can be found in Hoeksema et al. (2014). In this study, we used the random disambiguation for pixels with weaker linear polarization.

The VSM is a spectrograph-based instrument, which observes full line profiles of the Fe i 630.15 and 630.25 nm spectral lines, with a spectral sampling of 2.4 pm and pixel size of 1.0 × 1.0 (1.14 × 1.14 before January 2010) arcseconds over a 2048 × 2048 pixels field of view. To construct a full-disk magnetogram, the image of the Sun is scanned in the direction perpendicular to the spectrograph slit. At each scanning step, the spectra for each pixel along the slit are recorded simultaneously. Each scanning step takes 0.6 s, and a full disk magnetogram can be completed in about 20 min. The spectrograph slit is curved, which introduces geometric distortions to the image of the Sun. These distortions are corrected by shifting the position of each pixel in the final image to the closest integer position of the true pixel location in a round-Sun image. The maximum uncertainty in the position of a pixel does not exceed half-a-pixel, which is significantly smaller than typical atmospheric seeing for this groundbased instrument. The above correction procedure avoids ill-posed interpolation of full disk magnetograms, and it preserves the mapping of spectral information for each image pixel.

Similar to HMI, the observed profiles of Stokes *Q*, *U*, *V*, and *I* parameters are inverted using the VFISV code under the assumption of a standard Milne-Eddington stellar atmosphere. However, unlike HMI, VSM inversion includes the magnetic-field filling factor (*α*) as an additional fit parameter, which represents the fraction of each instrument pixel filled by magnetized plasma. For additional details about SOLIS/VSM inversion methods and pipeline, see Harker (2017). The 180° azimuthal ambiguity in the transverse field is resolved using the Very Fast Disambiguation Method (VFDM, Rudenko & Anfinogentov, 2014). The VFDM has an accuracy almost as good as that of the minimum energy method (used for HMI disambiguation), but is much faster. For a synoptic instrument such as VSM and HMI, the disambiguation is done automatically as part of the pipeline data reduction. The pipeline reductions are optimized for “a good answer most of the time, in time for the next dataset”, and in some cases may not return the best possible solution.

VSM data are from data processing level PROVER0 = 15.0511, which uses only one (Fe i 630.15 nm) spectral line for the inversion. In the following discussion we adopt the right-handed coordinate system (*B_r_*, *B_θ_*, *B_φ_*), which has been used in previous publications (e.g., Virtanen et al., 2019). Here the radial component (*B_r_*) is positive when pointing away from the Sun, the meridional component (*B_θ_*) is positive southward, and the zonal component (*B_φ_*) is positive westward.

## 3 Effects of noise in the transverse fields on the derived vector-field orientation

Let us now consider how properties of noise could affect the derived interpretation of the vector magnetic field. In all modern instruments, the derivation of vector magnetic fields is based on observations of full Stokes profiles in a selected spectral line sensitive to the effects of the magnetic field at the location of the formation of this line. Stokes *I* represents the total intensity of light. Stokes *V* is circular polarization (counter-/clock-wise), and Stokes *Q* and *U* represent two linear polarizations. The observed Stokes profiles are fitted by a model/synthetic line profiles in a process called “inversion”, and the properties of the magnetic field (and other parameters, such as Doppler velocity, temperature, magnetic filling factor etc.) are determined based on properties of the fitted line profiles.

The observed profiles of all Stokes parameters are affected by noise and other instrumental limitations. The photometric noise in Stokes *Q* and *U* is similar to that of Stokes *V*. However, the longitudinal (*B*_||_) field is related linearly to circular polarization, while the relation between transverse field and linear polarization is quadratic (e.g., see Eq. (18) in Stenflo, 2013 and Eqs. (12c) and (12d) in Jefferies & Mickey, 1991), which results in a lower signal/noise in the latter for the same underlying field strength. For example, in the weak-field approximation (Ronan et al., 1987; Jefferies et al., 1989; Jefferies & Mickey, 1991),

(1) $$B_{∥}\approx \frac{C_{∥}}{\alpha }\text{V}$$

(2) $$B_{⊥}\approx \frac{C_{⊥}}{\sqrt{\alpha }}\sqrt[{4}]{Q^{2}+U^{2}}.$$

Based on radiative transfer modeling of the Fe i 630.15 nm and assuming a filling factor of unity, *α* = 1 (the entire pixel is filled with a field), Stenflo (2013) estimated the coefficients of proportionality as *C*_||_ ≈ 29.4 and *C*_⊥_ ≈ 184. (The coefficients are model dependent, but *C*_⊥_ ⨠ *C*_||_ independent of a model).

As a result, the noise measured in horizontal fields is typically larger than the amplitude of noise in the longitudinal field by a factor of 10–25. Moreover, unlike noise in *B*_||_, which is distributed around zero, (*B*_⊥_ ± noise) is always positive (e.g. see Appendix A in Leka & Skumanich, 1999). For some specific magnetic configurations, this dichotomy may systematically distort the true inclination of the vector magnetic field.

**Fig. 2.**
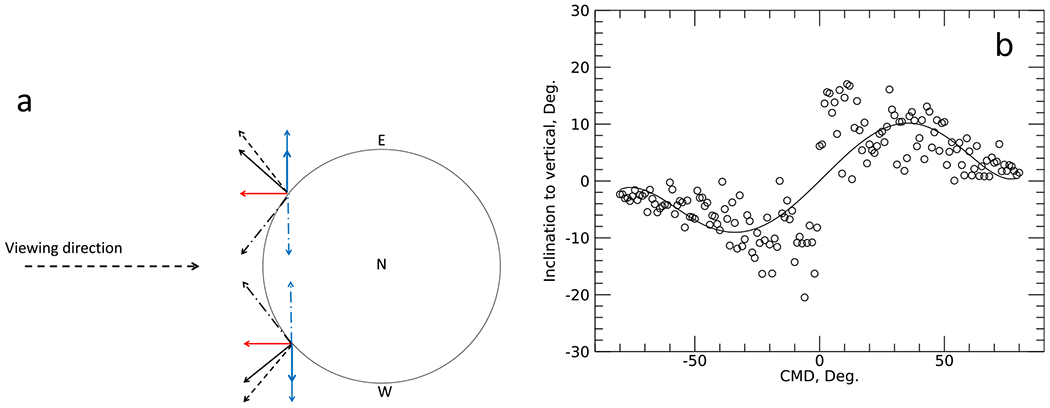
Effect of noise in the transverse field on the derived orientation of the vector field. (a) Black arrows represent the true vector field on the Sun (radial in this example). Red arrows represent the line-of-sight component and thick blue arrows are transverse components of the true vector field. Adding positive noise to the transverse field (thin blue arrows) makes the observed field (dashed arrow) systematically inclined in the direction away from the central meridian. Because of the 180° azimuthal ambiguity in the transverse field, the same transverse field may satisfy an alternative orientation (dashed-dotted arrow). The resulting orientation of the field vector is shown by a dashed arrow for both locations. Letters E, W, N mark approximate positions of solar East limb, West limb, and North as seen from Earth. (b) Difference between the true radial direction and vector orientation in the presence of noise, when azimuth disambiguation selects the most radial of two possible solutions (open circles). Solid line is a 6th degree polynomial fit.

Figure 2a shows a theoretical example, where the true magnetic field is expected to be radial (black arrow). Observed at disc center, a radial magnetic field is then determined by the observed line-of-sight component plus symmetric random noise in the azimuth direction, which is negligible on average. However, outside the central meridian as depicted in Figure 2a, a purely radial field contributes to both the line-of-sight component (red arrow) and the transverse component (blue arrow).

In the presence of noise, the orientation of the magnetic vector will be determined by the observed (*B*_||_ ± *δB*_||_) and (*B*_⊥_ ± *δB*_⊥_) components, where *δB* represents the amplitude of noise. Note that due to a quadratic contribution of Stokes *Q* and *U* to *B*_⊥_ (see, Eq. (2)), (*B*_⊥_ ± *δB*_⊥_) is always positive (by definition, transverse field is always positive, with or without noise).

For pixels situated East of the central meridian (upper part of Fig. 2a), the projection of the same (radial) vector would create a systematic non-zero transverse component, which in the presence of noise will only increase (due to *B*_⊥_ ± *δB*_⊥_ > 0). Due to the 180-degree ambiguity in the azimuth of the transverse field, the vector field would have two possible orientations, and if the more radial (black solid arrow) option is chosen, then the selected orientation of the magnetic field will have a systematic tilt in the direction away from the solar disk center. Figure 2b shows a difference between the true radial field and one with *δB*_⊥_ about 20 G added to the true transverse field. For this modeling exercise, we assumed that the true field strength of the vector field was 200 G, and *δB*_||_ = 0 (no error in the longitudinal field), and *δB*_⊥_ was randomly generated within the range of about 0–100 G, with a mean about 50 G and a standard deviation of 20 G. For pixels located closer to the disc center, we see an increase in scatter in the vector inclination relative to the true radial direction. Most importantly, there is a systematic inclination of the vector field in the direction away from the central meridian. For a field strength of 200 G and 20 G noise in transverse field, the inclination error could be up to 20° (Fig. 2b).

**Fig. 3.**
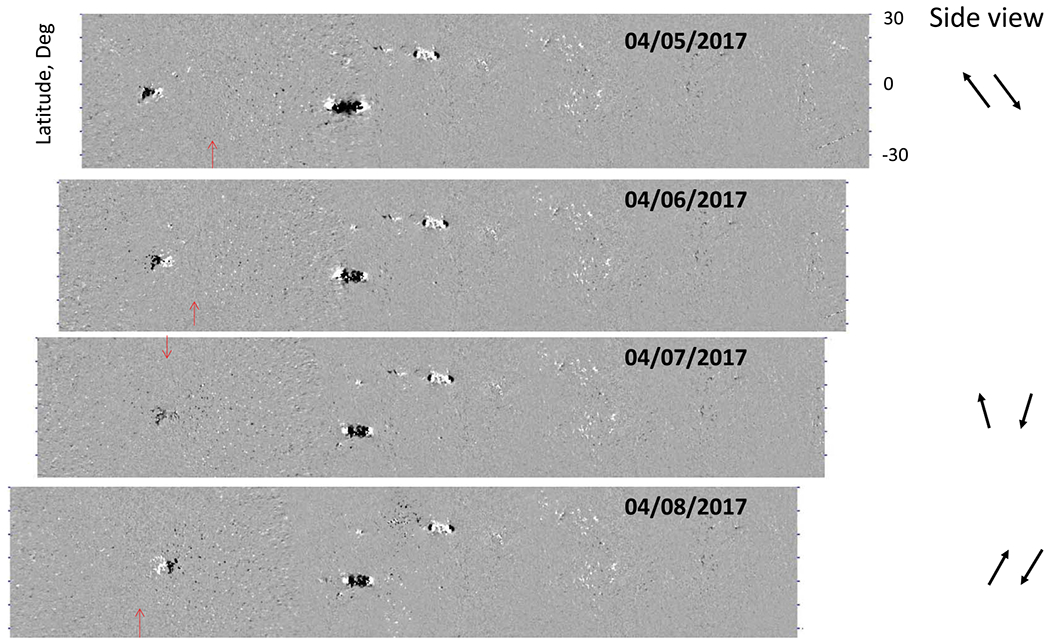
(Left) Changes in the pattern of the zonal (East–West) vector-field component of a small bipolar region over its disk passage. Four panels show the so-called near real time (NRT) synoptic maps. White/black halftones correspond to magnetic field directed towards the West/East. Each NRT map covers 360° in longitude (horizontal direction) and approximately ±30° in latitude (vertical direction). The most recent data are added onto the left side of synoptic maps. The dates of the most recent observations added to synoptic map are shown in the upper-right corner of each panel. For visual clarity, the synoptic charts are shifted to have the active regions aligned in the vertical direction. Small red arrows plotted in the left part of each panel correspond to the approximate location of the central meridian for the day of observations. Panels on the right show a schematic inclination pattern of the magnetic field vectors in a vertical East–West oriented plane (as if we were looking at an active region from the side).

Figure 3 demonstrates this effect in the disk-passage of a small bipolar region with negative leading and positive following polarity fluxes. It is clear that when this small active region is located to the East of central meridian, the horizontal magnetic field connecting the two polarities is directed westward in the leading (negative) polarity and eastward in the following (positive) polarity flux element. Only when the region is near the central meridian does it become clear that the magnetic field in both polarity fluxes is close to radial, with a very slight inclination in the direction away from each polarity. When the region is located West of central meridian, the pattern of the zonal field reverses as compared with its location East of central meridian. This behaviour is in perfect qualitative agreement with the explanation given in Figure 2.

**Fig. 4.**
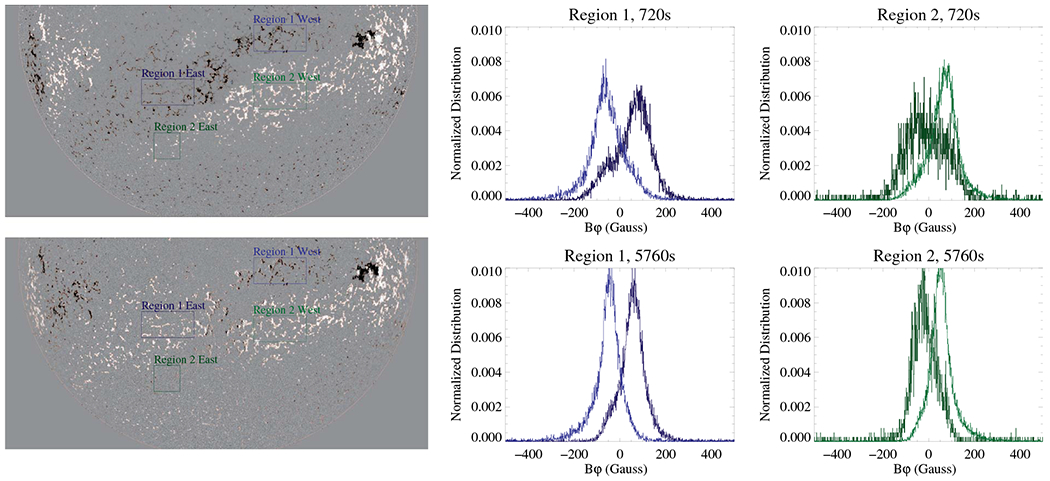
Upper-left: half-disk image of radial *B*_*r*_ field (white/black correspond to positive/negative polarity), scaled to ±200 G. Outlines show pixels with the minimum-energy disambiguation applied (confid_disambig keyword ≥ 60), which are included in the distributions shown in right columns. Lower-left: half-disk image of zonal *B*_*φ*_, scaled to ±100 G. Region 1 (blue boxes) corresponds to area of negative polarity flux in decaying flux region. Region 2 (green boxes) corresponds to a similar area but of positive polarity flux. Boxes outlined by lighter/darker color are located West/East of the central meridian. Middle column: distribution of the zonal (*B*_*φ*_) component of the magnetic field in the Region 1 for two (720 s and 5760 s) averaging. Right column: similar to middle column but for Region 2. Note: salmon (light pinkish–orange) contours that outline pixels with the minimum-energy disambiguation mask underlying black/white magnetic fields. To see both the magnetic field and the contours, the PDF figure needs to be magnified by 300% or more.

Let’s now consider whether lowering the noise level will mitigate this systematic effect. For this test, we selected HMI observations taken on 10 February 2015. That day had a good representation of various solar features (plage, sunspots) situated at different distances from solar disk center. For the test, we employed filtergrams that were processed using a much longer integration than normal (pipeline) magnetograms (5760 s vs. 720 s, courtesy Dr. X. Sun). Integrated (average) filtergrams then were inverted using standard VFISV code, and disambiguated using the minimum energy disambiguation method with default pipeline settings. The averaged magnetogram is centered at 19:12:00 UT, and is shown in Figure 4 (left). Despite significant time averaging, we do not see any obvious smearing of solar features. The *S/N* is improved when compared to the standard 720 s magnetograms, especially in areas with a weak polarization signal.

To test the effect of lower noise on the inferred direction of the magnetic field vector in large-scale weak-signal areas, we select two decaying plage regions that extend across the central meridian, and sample the *B_φ_* on either side (light/dark blue and green boxes in Fig. 4). We assume that the field vectors in these structures should not vary across the central meridian per se. Figure 4 (right) shows the distribution of the zonal (East–West) magnetic field in the area of negative (blue colors) and positive (green colors) polarity flux. The distributions show a clear offset: for pixels situated East of central meridian, the mean is positive, while for pixels in the Western hemisphere, it is negative. Such an offset supports the notion that a magnetic field situated in a region of decaying magnetic flux in the Eastern hemisphere, on average, shows inclination patterns away from the solar central meridian. Using observations with a lower noise level (5760 s) makes the distributions narrower, but the offset is still present. This behavior is in agreement with the explanation presented in Figure 2.

## 4 Effects of azimuth disambiguation and filling factor

For a simplified case of *B_θ_* = 0, shown in Figure 2a, the transformation from longitudinal and transverse components, measured in the image plane at a heliographic longitude (central meridian distance) *ϕ*, to heliographic components *B_r_* and *B_φ_* can be written as

(3) $$B_{r}=B_{∥}\cos\;\phi \pm B_{⊥}\sin\;\phi \;=\frac{C_{∥}}{\alpha }V\cos\;\phi \pm \frac{C_{⊥}}{\sqrt{\alpha }}\sqrt[{4}]{Q^{2}+U^{2}}\;\sin\;\phi$$

(4) $$B_{\varphi }=-B_{∥}\sin\;\phi \pm B_{⊥}\cos\;\phi \;=-\frac{C_{∥}}{\alpha }V\sin\;\phi \pm \frac{C_{⊥}}{\sqrt{\alpha }}\sqrt[{4}]{Q^{2}+U^{2}}\;\cos\;\phi .$$

For this simple configuration, the ± ambiguity in equations (3) and (4) is resolved by requiring the two components to have the same sign in equation (3) and consequently opposite signs in equation (4). The first component (Eq. (4)) is positive East of the central meridian (*ϕ* < 0), and it is negative West of the central meridian. The second component is always positive. For the case when the field is mostly radial

(5) $$\left|\frac{-C_{∥}V}{\alpha }\right|\gg \frac{C_{⊥}\sqrt[{4}]{Q^{2}+U^{2}}}{\sqrt{\alpha }}.$$

Under this condition, the sum of the two components, which represents the sign of *B_φ_*, will be positive East of the central meridian and negative to the West. However, in the case of a mostly horizontal field

(6) $$\left|\frac{-C_{∥}V}{\alpha }\right|\ll \frac{C_{⊥}\sqrt[{4}]{Q^{2}+U^{2}}}{\sqrt{\alpha }}.$$

The sign of *B_φ_* will be always negative, independent of pixel location relative to the central meridian. This is in qualitative agreement with the observations mentioned in Section 1 that the disagreement in the sign of the zonal (*B_θ_*) and/or meridional (*B_φ_*) components of HMI and VSM observations was only observed in pixels with weaker linear polarization signals. In pixels with stronger linear polarization, the two instruments were in agreement with each other with respect to the sign of *B_φ_* and *B_θ_* components. Equations (5) and (6) suggest that the effect of sign reversal in *B_φ_* could also depend on magnetic filling factor *α*. For some amplitudes of $\left|-C_{∥}V\right|\text{ and }C_{⊥}\sqrt[{4}]{Q^{2}+U^{2}}$ the inequity in Equation (5) could change its sign if *α* is changed from being less then one to unity. Appendix provides an example of a test done with SOLIS/VSM data, which demonstrates that for *α* < 1, *B_φ_* in three flux areas reverse their sign when the area crosses the central meridian. When *α* is set to unity, the same areas do not exhibit a sign reversal.

## 5 Discussion

We provide simple arguments and some observational evidence that a dichotomy in properties of Zeeman polarization arising from longitudinal and transverse field components, and our ability to interpret these signals especially in unresolved structures (non-unity filling factor), combined with the azimuthal disambiguation may lead to erroneous conclusions about the orientation of the vector magnetic field, particularly in areas where the polarization signals are weak. The systematic patterns may depend on the amplitude of noise and whether the magnetic filling factor is resolved or assumed to be unity, and thus, observations from different instruments could result in slightly different orientation patterns (e.g., inclination of vector fields towards the solar poles or towards the solar equator). Pixels with stronger polarization signals are less affected (e.g., in sunspots, where *α* is relatively large, and where Stokes *Q*, *U*, and *V* are typically strong), and thus, we expect that only pixels with weaker polarization signals will show the effect described above. Indeed, comparison of observations from SOLIS/VSM and SDO/HMI show a good agreement between the two instruments in areas of strong fields (e.g., sunspots), while in some areas of weak fields (e.g., plage), we see the zonal (East–West) and/or meridional (North/South) components having opposite sign. The amplitude of the effect will depend on the specific orientation of the magnetic field vector relative to line-of-sight, and thus the impact of this effect could vary across the solar disk in a somewhat complex way, making it difficult (or perhaps impossible) to correct, since the signal inherently originates from an unknown magnetic configuration. For an underlying radial-field orientation, the effect could be mitigated by equalizing the amplitude of the noise between the inferred longitudinal and transverse field components. However, given the significant differences in noise levels between inferred *B*_||_ and *B*_⊥_ such noise equalization could be done only at the expense of increasing noise in the longitudinal field measurements (e.g., by using a modulation schema allowing much longer integration time for states corresponding to Stokes *Q* and *U* measurements). A test conducted using existing HMI data shows that simply improving *S/N* for both transverse and longitudinal fields does not completely eliminated the problem; while the amplitude of a systematic inclination decreases slightly, the overall effect still remains. We note that the systematic bias in the horizontal component of the magnetic field may, in principle, lead to the effects similar in appearance to ones that we discuss here. While our simplified example discussed in Section 4 uses filling factor, it might be extremely difficult or even counterproductive to model the exact magnetic and non-magnetic contribution in pixels outside sunspots. The methods used to estimate the filling factor may inadvertently introduce additional errors related to the unknown difference in line profiles between magnetized and non-magnetized atmospheres in each pixel. The effects of the contribution function in spatial direction (e.g., whether non-magnetized component is predominantly located on one side of a pixel or it is uniformly distributed around it as in Harker, 2017) are also unknown. Perhaps, some of these issues may be addressed via spatially coupled inversion of spectro-polarimetric image data as in van Noort (2012). That technique, however, requires the knowledge of the point-spread-function (PSF), which could be achieved either by measuring the atmospheric seeing during the observations (van Noort, 2017) or using adaptive optics. Thus, the method is not applicable for the existing SOLIS/VSM observations.

## Figures and Tables

**Fig. A.1.**
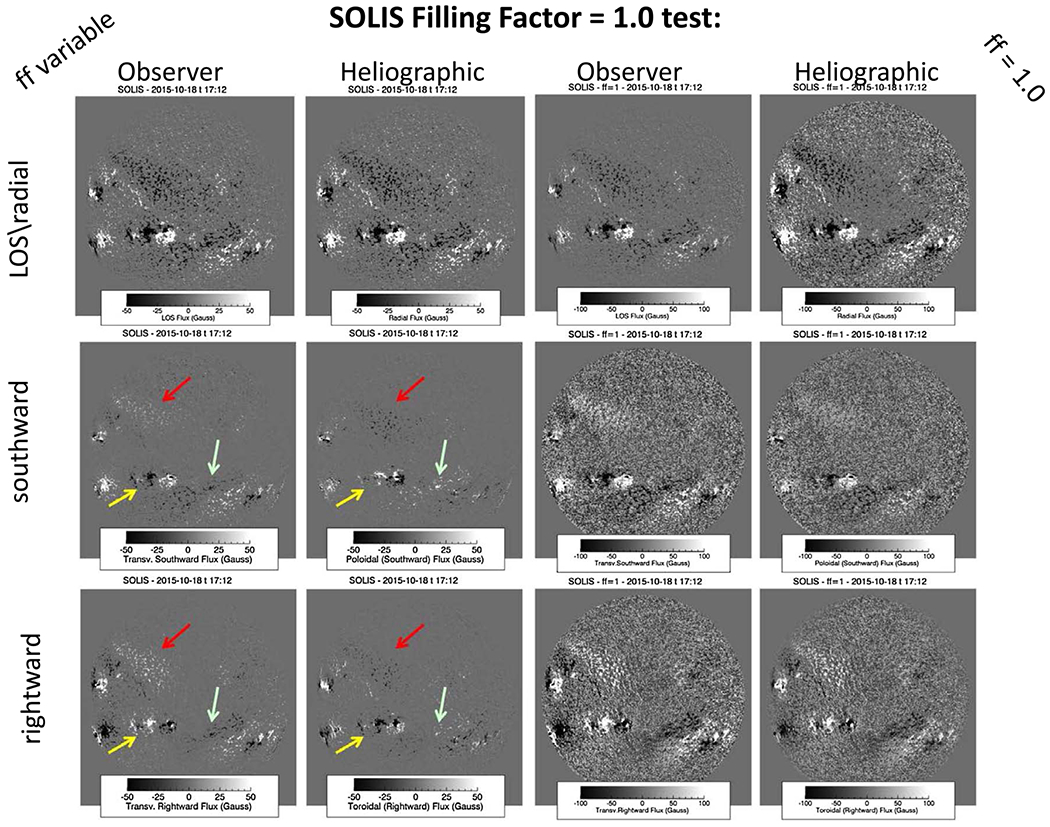
Full disk images of *B*_*r*_ (upper row), *B_θ_* (middle) and *B_φ_* (low row). First and third columns show the vector field components in image coordinate system, while second and fourth columns are in the heliographic coordinate system. Three examples are marked by arrows of different color. Setting *α* to unity emphasizes pixels, which otherwise are below the noise threshold.

## Appendix

### The effect of magnetic fill factor on the horizontal components of magnetic field

Figure A.1 provides an example of a test done with SOLIS/VSM data, which demonstrates that for *α* < 1, *B_φ_* in three flux areas reverse its sign when the area crosses central meridian. When *α* is set to unity, the same areas do not exhibit the sign reversal.
